# Fascial Manual Medicine: A Continuous Evolution

**DOI:** 10.7759/cureus.71442

**Published:** 2024-10-14

**Authors:** Bruno Bordoni, Allan R Escher

**Affiliations:** 1 Physical Medicine and Rehabilitation, Don Carlo Gnocchi Foundation, Milan, ITA; 2 Oncologic Sciences, University of South Florida Morsani College of Medicine, Tampa, USA; 3 Anesthesiology/Pain Medicine, H. Lee Moffitt Cancer Center and Research Institute, Tampa, USA

**Keywords:** fascia, manual therapy, myofascial, osteopathic, osteopathy, quantum biology, quantum physics

## Abstract

From the perspective of fascial manual medicine (FMM), the body should not be considered as a set of compartments, but as a functional continuum, where most of the tissues (considering embryology) are fascia. The cells that make up the fascia can use multiple strategies to communicate, with neighboring cells, with the tissue to which they belong, and with the entire body, thanks to biochemical (microscopy) and electromagnetic (nanoscopy) possibilities. These multiple capacities to send and receive information make the border or layer of the different tissues seem absent. All the manual techniques that profess to be the only ones that work on the patient's symptoms, dictating a standardized manual procedure that all patients should undergo, represent a clinical deviation. Likewise, thinking that the manual approach can provide biomechanical stimuli only to a single specific structure or layer is a conceptual error. This narrative review briefly reviews the history of fascial-related nomenclature and how the fascial system is currently considered, posing new reflections on how the fascial continuum could be conceived by practitioners who apply FMM in the clinic, such as osteopaths, chiropractors, and physiotherapists.

## Introduction and background

The function and cohesion of biomolecular systems in the human body are based on the theory of quantum mechanics, where biological self-organization occurs through cellular “awareness” and a process of entanglement [1]. The propagation of a nervous impulse, for example, is the mirror of the speed of energy exchanges between electrons, which come from elementary particles such as fermions and bosons; this exchange of sub-atomic energy creates a bond that lasts forever, regardless of time and space (entanglement) [2]. Furthermore, the response of each single cell to the information it perceives is translated by multiple interconnected intracellular systems, creating a “nano-brain”; the cell behaves by weighing the stimuli it receives in a sort of consciousness [3]. The constant passage of sub-atomic information creates electromagnetic vibrations/waves, which constantly keep the cellular structures (such as the membrane, the cytoskeleton, and the various proteins) in a constant vibratory state [4]. Vibration is information (form), while form is the response to information (function). According to quantum optics, each cell is aware of what happens to all the other cells. This would allow the formation of memory [4].

Memory (form and function: vibrations and responses) is transmitted from the body to the surrounding environment and vice versa, that is, we are not detached from the environment in which we live, and we can influence the environment in turn. This happens because matter as we understand it is the response to infinite exchanges of energy between elementary particles, which form atoms, cells, tissues, and the body. Cellular memory is an intertwining of vibrations and mirror responses. The fact that entanglement exists (a bond that lasts forever), and independently of time and space (distances), memory is needed for evolution and survival [5-7]. According to quantum physics, a cell would also be able to anticipate responses, before the information arrives, as each particle behaves as the particle it is in contact with (quantum coherence), thanks to the phenomenon of entanglement. This mechanism at the basis of life implies an implicit systematic learning [8]. The human body is a quantum coherence; life reverberates in the infinitesimal.

Quantum biology is a new scientific discipline that combines physics, chemistry, and biology: “This is an exciting and emerging scientific exploration, paving the way for innovative discoveries and developments that will revolutionize healthcare” [9]. Our understanding of the function of the human body and how it responds to manual treatment demands is based on theoretical models, which fail to take into account that every act of manual medicine acts not only at the macroscopic level but also at the microscopic and nanoscopic levels. This means that respecting the dictates of quantum biology, in theory, we can act on the multidimensionality of the body with gentle manual approaches, and that knowledge of the simple macroscopic anatomical description is not sufficient to help the clinician who uses fascial manual medicine (FMM) to understand the patient's body function.

Quantum physics predicts that a particle has a non-zero probability of spontaneously crossing a potential energy barrier even when its kinetic energy, according to classical mechanics, would not allow it. This is the quantum tunnel effect that has received countless experimental confirmations. Well, it would seem that enzymes exploit this effect to transfer spatially and energetically separated subatomic particles, such as electrons and protons, from one end of the molecules involved to the other, thus accelerating the related chemical reactions. Even more interesting, from a clinical perspective, is the hypothesis that the tunnel effect itself is responsible for genetic mutations, favoring the physical movement of particles from one position to another of one of the nitrogenous bases that make up the nucleotides of DNA and RNA. The change in position causes, in fact, a different arrangement of the hydrogen bonds with the complementary base, determining a mutation. Previously, we have published articles discussing the importance of cellular microstructure in understanding how tissues interact and function, highlighting the importance of the microscopic in understanding the macroscopic [10,11].

Every cell is a three-dimensional structure, which contacts (directly and indirectly) and influences all other cells and tissues. For example, indirect contacts micro ribonucleic acids (miRNAs) are a class of small non-coding RNAs (18-25 nucleotides), which regulate gene expression for body homeostasis [12]. They are produced from DNA and translated in the cytoplasm and then out of the cell membrane; they can travel within extracellular vesicles [13]. The latter are small bags filled with multiple biochemical information and sent by each cell; like a postman, it influences the function of target cells, near or far [14]. To give an example of direct contact, each cell can form tunneling nanotubes (TNTs). The latter connect cells several micrometers apart and are cytoskeleton protrusions made up of actin and tubulin filaments [15]. These contractile structures are not permanent and are replaced over time by other TNTs, allowing the transport of numerous biochemical and electromagnetic information between distant cells. From the point of view of surgical medicine, it is essential to know the different tissue layers (macroscopic), both to avoid iatrogenic damage and to reach (for example with drugs) the anatomical area sought [16,17]. Likewise, for anatomists who study the different anatomical areas, it is essential to be able to separate each single layer (macroscopic), to obtain a nomenclature that is shared and useful in surgical practice [18,19]. From the point of view of FMM, the body is not considered as a set of compartments, but as a functional continuum, where most of the tissues (considering embryology) are fascia [20,21].

The article briefly reviews the history of the nomenclature and how the fascial system is currently considered, raising new considerations on how the fascial continuum could be conceived by practitioners who apply FMM in the clinic, such as osteopaths, chiropractors, and physiotherapists.

## Review

The path of the concept of fascia in history

We searched the literature (PubMed) for information that traced the history of the meaning and nomenclature of the fascia; furthermore, in the continuation of the text, we reviewed some concepts (always PubMed) that highlight a new vision of the fascial system, such as quantum biology. What do we mean by “history” of fascia? History has provided a wealth of knowledge about the fascial continuum; using this knowledge allows us to improve clinical practice in the present.

The Latin feminine term that we commonly use (“fascia”) derives from the Greek word “taenia”, which word implies a ribbon-like structure, bandage, threads, or thin strips [22]. From Latin we also find the plural of fascia (“fasce”), that is, “fasciae”. We do not know, however, if this terminology was associated with the anatomical field. In the Persian Empire of the Middle Ages, between the eighth and thirteenth centuries AD, the term fascia was already present in the field of anatomical dissections. According to the anatomists of the Persian era, the fascia was associated with the nervous system and considered as a path of pathological or physiological stimuli, therefore an active tissue in maintaining the health of the patient [23]. It is a tissue, therefore, visible to the naked eye, a concept still used by modern anatomists [22]. The books of medieval Persian scholars were known in Western Europe from the sixteenth century. And in fact, in the sixteenth century the word fascia was transported into the English language, but always related to objects and not to anatomy [22].

In 1615, the term "fascia" appeared in a writing by Dr. Crooke, describing some anatomical structures that today are identified as thoracolumbar fascia and fascia lata, highlighting how these body areas appeared as membranes. Crooke was the first Western anatomist to use the term fascia in an anatomical context [22]. In 1694, Dr. Cowper described the fascia groups, including the muscles of the forearm, and identified the tendon of the biceps brachii as a fascia; in his written dissertations, he revealed that the muscles of the lower limb are covered by a membranous fascia. Furthermore, he gave an important concept, namely that the fascia lata (as we know it today) connects the hip to the ankle, maintaining the muscle tissues in a correct position [22].

Seventeenth-century writings described fascia as a membrane that covers, protects, and connects different layers. Crooke emphasized that this material is different depending on its width, position, and shape and that each layer is crossed by vessels and nerves. He highlighted that such membranes are found throughout the body, acting as a scaffolding, and raised an extraordinary reflection, namely, that these fasciae act as organs of sense of touch. In 1651, he drew up the first taxonomic classification of fasciae, which is not so different from the most well-known current classifications [22]. According to another seventeenth-century anatomist, Dr. Collins, fascia has a less dense structure than skeletal structures; furthermore, he described in his writings that this tissue had a greater capacity for elasticity, precisely thanks to the intrinsic distribution of the filaments that composed it [22]. These membranes appeared, according to Collins, to be closely connected to the tissues they covered, and like Crooke, he pointed out that such membranes allowed the various body structures to maintain their position.

In the eighteenth century, the term fascia was used in the anatomical field, but it mainly had the meaning of indicating a membrane or an aponeurotic structure, a tendinous expansion, fibrous sheaths, or something that wraps [22]. In 1751, Dr. Haller described the membrane fascia made up of fibrils, organized in such a way that it was able to carry out and undergo large movements. With Dr. Simmons in 1780, the interest was still oriented toward the microscopic, describing the fascia as a web of communications, which thickened or decreased its content depending on its function, constituting a single entity; he also described the presence of water inside the fascial network [22]. The microscopic level of the fascia was seen in this century as a gelatinous environment. Therefore, concepts such as network, sense organ, and continuity emerged in two centuries in Western medicine.

In the 19th century, the term fascia was used in a broader percentage. In 1801, anatomy terms appeared to describe five specific muscle areas with the term fascia, such as fascia lata or lumbar fascia. An American medical journal from 1814 described a trauma to a soldier of the first battalion of the sixty-second regiment, who was subjected by Dr. Mackesy to a deep surgical cut, highlighting that he had succeeded in dividing the deep fascia of the leg [24]. In 1839, other body districts took on the word fascia in medical dictionaries (eight areas in total); in 1876, 26 anatomical areas described with the term fascia appeared, arriving in 1892 with 231 fascial anatomical parts. This exponential growth depended on the discovery of new, previously unknown fascial areas [22]. The classification of these fasciae would depend on their location (superficial, deep, cervical, lumbar), morphology (triangular, funnel-shaped), hypothetical function (for the insertion of muscles), macroscopic characteristics (loose or denser), and proximity to other anatomical structures (intermuscular, renal fascia). Some authors such as Bichat wrote distinctions between the term fascia/fasciae (long and thicker), which was associated with muscle tissue, and the term membrane (thin), which was described as an organ. Furthermore, he stated that, despite the apparent diversity of membranes, they connected all tissues [22]. In 1800, the concepts of fascial layers, of fascial membrane as an organ, and of a continuous connection were reaffirmed.

Another concept that emerged from the anatomy books of the 19th century, from 1851, was that the loose and fibro-areolar fascia (dermis) was the superficial fascia below the epidermis and that the deep fascia concerned the musculature. The epidermis was excluded from the concept of fascia, even though the latter had the viscoelastic properties of any connective tissue [25,26]. This “axiom” can be found in Gray's book of 1858 on pages 186-187 [22]. Again, Bichat states that at the cellular level, this assembly of wavy and thin filaments can be found throughout the body.

In the twentieth century, the nomenclature of fascial topography increased exponentially and in greater detail, coining a terminology endorsed by anatomy organizations, such as the Federative Committee on Anatomical Terminology (FCAT), and the International Federation of Associations of Anatomists [22]. Not only did anatomists impose a nomenclature, but terminology related to surgical activity began to appear, such as fascial planes, fascial spaces, and fascial systems. What has been recognized over the past century is the fact that the fascia is continuous in the body, and it is not possible to recognize an end or a beginning [27].

In the twenty-first century, publications on the topic of fascia increased, involving not only anatomists and surgeons but also other figures related to patient health, such as physiotherapists, osteopaths, chiropractors, and specialists in other clinical fields. Thus, we find the Fascia Research Congress in 2007 and the Foundation of Osteopathic Research and Clinical Endorsement (FORCE) in 2013. FORCE tries to put the most up-to-date scientific concepts into daily clinical practice, to make FMM more concrete. In this 20-year period, the fascia began to connect to areas of health and disease, trying to associate the fascial system with dysfunctions that could lead to pathologies, with some new terminologies, both from a macroscopic and microscopic point of view [22]. The fascia is associated with the capacity for bodily movement, the ability to promote tissue repair, and the capability to influence aging and systemic inflammatory status and is one of the causes of the presence of chronic and acute pain [28].

We find new concepts by connecting manual therapy to fascia. Fasciology is a new term that relates the concept of traditional Chinese medicine, such as meridians in acupuncture, and the fascial system, according to Dr. Yuan [28]. Another example is the term fasciatherapy, which is given to gentle manual approaches, with non-invasive and non-instrumental therapeutic treatments [29]. The body is conceived as a set of myofascial chains or meridians, capable of influencing biomechanics and helping or slowing down movement depending on the presence or absence of dysfunctions of these links [30]. Other groups placed a copyright on the name given to manual fascial work, limiting conceptual freedom for the first time, which is a serious precedent for the limitation of scientific freedom.

Currently, there is a lack of a univocal nomenclature and understanding of what fascia is [31]. A scientific tool that could help to understand how to intend fascia is embryology. Embryology is the basis for understanding the function of tissues; understanding the function makes it easier to create a more appropriate nomenclature [32,33].

Fascia embryology

The connective tissue is the fascia. Fascia can be divided into connective tissue proper (loose or areolar, and dense), and specialized connective tissue (solid and fluid fascia) [20,34]. All these classifications include the presence of collagen, fibroblasts, and telocytes [20]. All tissues understood as connective fascia include common characteristics, such as viscoelasticity and the ability to produce movement [20,35,36]. Adipose tissue, considered a connective tissue, contains collagen fibers (particularly type VI) [35,37,38]. Fibroblasts, which synthesize collagen, produce movement, with an average peak contraction every 84 seconds approximately [39,40]. Adipocytes also have viscoelastic properties [41]. Likewise, osteocytes have contractile properties and viscoelastic capacity; collagen is interconnected with osteocytes [42-44]. Blood cells such as red blood cells and platelets are viscoelastic structures and capable of actively changing morphology [45-48]. Another characteristic shared by the fascia is the embryological phylogeny. The mesenchyme is a tissue immersed in an extracellular matrix, made up of loose cells, fluids, and proteins; this tissue organization allows cells to easily migrate from the main embryonic layers from the mesoderm and the neural crests of the ectoderm [49]. During gastrulation (after the third week of development) the mesenchymal cells or epithelia lose their adhesive capacity and can migrate from the layers from which they derive. This process is known as epithelial-mesenchymal transition. The mesodermal mesenchyme and the ectomesenchyme will give rise to the connective tissues of the body [50,51]. The connective tissues of the body can be classified into solid tissues (connective tissue proper, fat, bones, cartilage, meninges, tissues representing the vascular and lymphatic pathways, dermis, and joint capsules), and fluid tissues (blood, lymph, and cerebrospinal fluid) [20,31].

**Figure 1:**
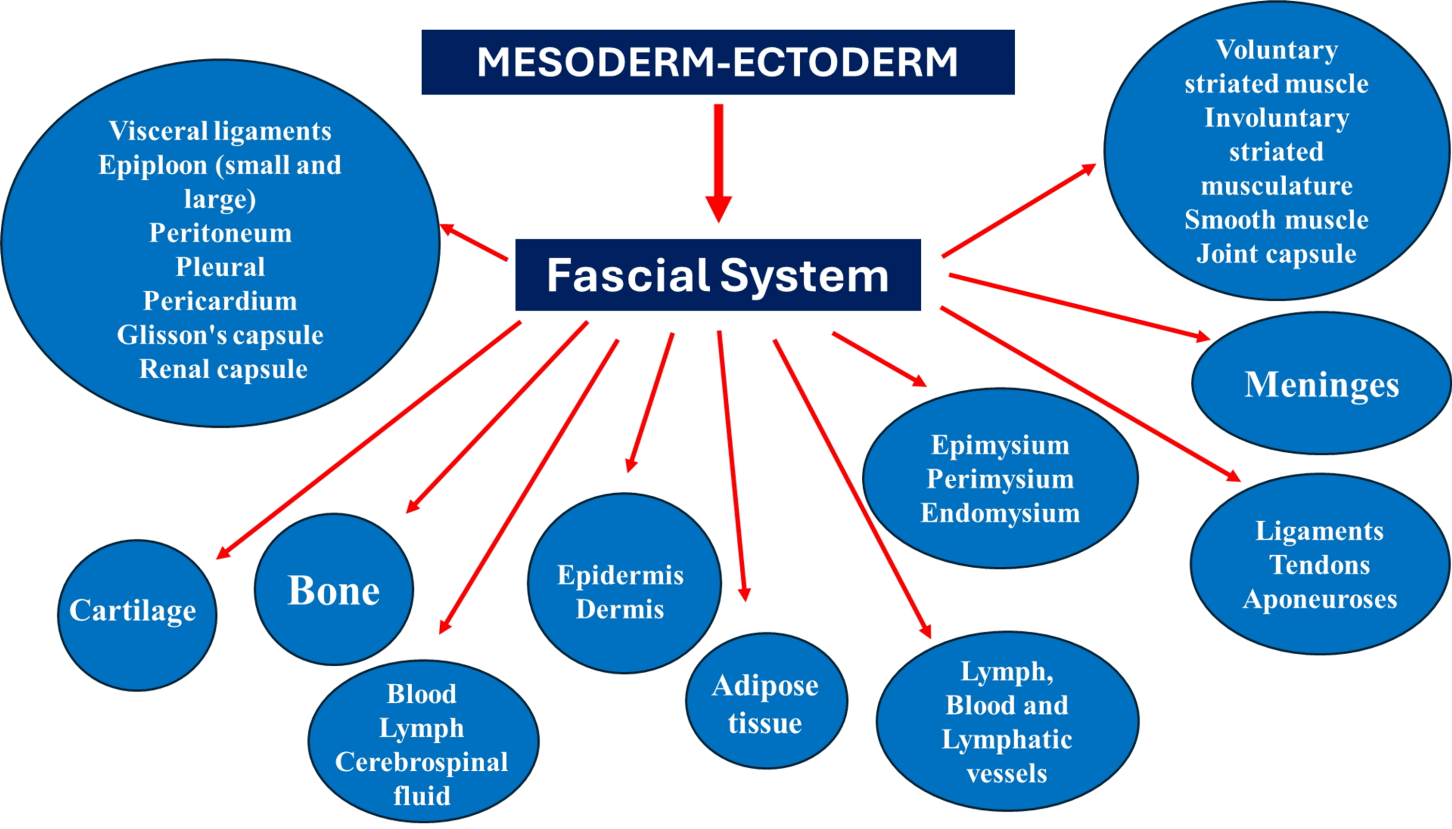
The diagram illustrates what our non-profit organization (FORCE) considers connective tissue. The image was previously published in the Cureus Journal of Medical Science [20]. FORCE: Foundation of Osteopathic Research and Clinical Endorsement

We have known since 1888 that the neural crests give rise to the connective tissue for the cranial and cervical-cranial area, while the mesodermal layer gives rise to the connective tissue for the rest of the body [52]. In some muscles of the cervical-cranial tract, there is a phylogenetic mix of connective tissue, such as the trapezius muscle and the sternocleidomastoid muscle, without anatomical discontinuity or recognizable limits from a macroscopic or microscopic point of view [20,21]. In our previous works, we considered other structures that could be considered connective tissues starting from the embryological origin (mesoderm, ectoderm), such as the voluntary and involuntary smooth muscle fiber (Figure 1) [20,21]. The functional unit of the muscle fiber performs cyclic contractions in the absence of a specific electrical order, such as fibroblasts, myofibroblasts, and smooth muscle cells [53-55]. Muscle fiber and sarcomere possess a viscoelastic capacity; this property makes muscle tissue a component of the fascial continuum [56-60]. A connective tissue could be considered as a structure that moves, creates action, and supports the continuity of movement.

FMM in the present

We know that fascia or connective tissue has a double embryological phylogeny, like ectoderm and mesoderm. We know that what is considered fascia has the same characteristics of viscoelasticity and active movement. We know that every cell communicates with every cell next to it and with all body tissues. Communication presupposes bi-directional exchanges of information capable of changing or maintaining a physiological status or leading to a disease condition. To give some examples, miRNAs at the brain level can influence behavioral and cognitive capacity, if the stimulus that produced them is not salutogenic [61].

In addition to TNTs, cells can contact other structures by creating cytonemes, which are specialized protrusions or filopodia that can fuse with an adjacent membrane; cells can create smaller protrusions or buds from the plasma membrane, again to transfer bioactive substances and/or more complex molecules [62]. Cells can communicate using electromagnetic waves, effectively making each layer non-present and non-limiting [63]. The ability to process, send, and receive non-chemical information between cells is an event known since the 1920s; Dr. Gurwitsch called this action "mitogenic radiation", as the informational exchange without chemical intervention stimulated cellular maturation [63]. No cell alone can survive, as without information it does not adapt and dies.

Technology makes us forget the importance of using hands, palpation, and evaluation of the patient's tissues [64]. The repetitive habit of the clinic and the desire to always look for something new in research make us forget that some information should be eliminated or changed. For example, the epidermis has viscoelastic characteristics, the cells that constitute its structure (keratinocytes and melanocytes) contract, and it derives from the ectoderm [65-67]. Excluding the epidermis from the concept of fascia comes from 1851 when the notions of embryology and microscopy were severely limited. Probably, the epidermis should be included in the concept of fascia.

FMM does not work on the layer, area, or a specific tissue, because from a functional, physiological, and quantum point of view, the body functions as a unit; each touch is felt by the whole body and not only by the layer, area, or a specific tissue. From a functional body perspective, a regional inflammation status involves the whole body; the nervous system records this immune information and can be negatively affected if this biochemical information becomes chronic. Over time, this local inflammation can transform into a systemic disease [68]. Furthermore, the entire system retains the memory of what happens to the entire body, with the risk of pathological dysfunction recurring [69].

All manual approaches and techniques that describe a standardized and equal treatment path for each patient are a clinical aberration; it is the clinician who must adapt to the patient based on subjective needs. If the concept that only a specific manual treatment is valid for everyone was real, manual medicine, pharmacology, and surgery would not exist. Clinical failure would not exist.

Each component that constitutes the connective tissue can interact systematically and influence the health status of the person. Adipose tissue, if in excess (body mass index greater than 30 kilograms per square meter), can stimulate a systemic neuroinflammatory environment, with the constant production of inflammatory cytokines (interleukin-1ß, interleukin-6, interferon-γ, tumor necrosis factor-alpha, and monocyte chemoattractant protein-1) [70]. The latter will be able to cross the blood-brain barrier and destroy the systemic immunological balance.

Bone tissue communicates with the entire body system. For example, the bone secretes osteocalcin and fibroblast growth factor 23 (FGF-23), whose molecules can cross the blood-brain barrier, affecting the function of the central nervous system, cognition, mood, and the ability to store data. This creates a bone-brain axis [71].

Muscle fibers produce many biochemical substances in a paracrine (and autocrine) fashion, for the entire body system. Muscle secretes myokines (interleukin-6, 8, 15, follistatin-like-1), which can influence the systemic immune response and the general metabolic status, as well as endothelial health and the regenerative capacity of the muscles themselves [72]. A muscle-brain axis is created for systemic and cognitive health [73]. Smooth muscle fibers, if removed from their homeostasis, are stimulated to produce pro-inflammatory cytokines and chemokines, modulating the local and systemic immune response [74,75]. The task of the FMM is to find a manual path that allows the tissues to find more space for movement; in this way, it improves the flow of fluids, improves afferents, and recreates a set of more physiological efferent information for the tissue itself and the body system. FMM awakens the intrinsic salutogenic memory of tissues [76-80].

Andrew Taylor Still, a doctor of osteopathic medicine (DO), wrote: “The fascia is the place to look for the cause of disease and the place to consult and begin the action of remedies in all diseases” [81]. We could say that we are a “walking fascia”.

Thus, the clinician who deals with FMM should increase palpatory ability, always subjectivize the chosen treatment, and always keep active their intellectual curiosity to welcome new information that may derive from other scientific disciplines. Stopping only on one's own knowledge is not adaptation, as adaptation is improvement; this last concept happens only if one is ready to change scientific perspectives [82,83].

We reiterate that the body is a unit and that it does not work as a set of uncoordinated segments [81,82]. FMM acts on the whole body, even if it must be worked manually starting from a specific area. Furthermore, the fascial continuum should consider some characteristics that unite the tissues, such as viscoelasticity, active movement, and the embryological derivation of the connective tissue. Everything that moves in the human body is fascia.

Quantum mechanics is now over a century old, since that autumn afternoon in 1900 when Max Planck discovered that energy exchanges in the phenomena of emission and absorption of electromagnetic radiation occur in a discrete form, not in a continuous form as the classical electromagnetic theory claimed. It is still a relatively young scientific field in which theoretical physicists, chemists, and molecular biologists from all over the world are working on the collection of experimental data and speculative intuitions with the common intent of shedding light, through quantum mechanics, on biology. Further studies are needed to deepen our vision of how to conceive the fascial tissue in its functional entirety, improving the clinical approach and inserting the new discipline of quantum physics into a common vision.

Future tools to observe the human body

Current instrumentation does not always capture what is happening in the nanoscopic realm, and this means losing further information useful for understanding how the fascial continuum behaves and how the clinician can improve the approach to the patient with the FMM. Furthermore, a greater understanding would allow for a better nomenclature of the fascia based on function and not only on topography. A tool that will allow us to observe the human body, measuring single molecules is single-molecule localization microscopy or Förster resonance energy transfer. It is possible, for example, to measure the stoichiometry of biomolecular interactions and intra- and intermolecular distances. Furthermore, it is possible to study the frequency and duration of binding events or qualitatively interpret the data to study transient conformational changes of macromolecules [84,85]. Another strategy to improve our understanding of each individual component of the human body is optoacoustic (photoacoustic) imaging. The latter is a rapidly developing hybrid modality of biomedical imaging. It is a non-invasive technique that combines the high contrast of optical imaging with the spatial resolution of ultrasound imaging [86]. Structured light microscopy is a technique that allows double the resolution compared to traditional confocal microscopy, with a resolution of 100-130 µm [87].

Measuring and observing the nanoscopic environment is a fascinating field of research. A recent paper describes the use of nanoscopic imaging of the hydrated nanoparticle-ligand interface. The study observed the nanoscopic behavior of molecules and ion channels in a fluid environment, i.e., an environment that most closely resembles a biological/living environment [88]. The future of this new tool will allow us to understand biology starting from the nanoscopic [89]. The instrumentation will be both for the clinic and to better understand manual medicine.

## Conclusions

From the perspective of FMM, the body is not considered as a set of compartments, but as a functional continuum, where most of the tissues (considering embryology) are fascia. The cells that compose the fascia can use multiple strategies to communicate, both with neighboring cells, with the tissue to which they belong, and with the whole body, thanks to electromagnetic waves, miRNAs, extracellular vesicles, TNTs, cytonemes/filopodia, making the concept of layer insufficient to fully understand the functional entirety of the body. The clinician who uses manual therapy, considering current scientific knowledge, should consider other tissues as part of the fascia, such as the epidermis and the striated and smooth muscle contractile tissue. The manual approach should never be standardized, but subjective, following the clinical needs of the patient. We are aware that research must make further efforts to improve the understanding of what the fascial continuum is and how it works. The current literature highlights the function of the fascial continuum mainly from the aspect of the macroscopic structure, partly from the aspect of the microscopic structure, and almost none from the aspect of the nanoscopic component; this last one is the new frontier of research.
